# Supplementary material for: Molecular Characterization, Expression Profiling, and SNP Analysis of the Porcine RNF20 Gene
Source: Animals (Basel). 2020 May 20;10(5):888. doi: 10.3390/ani10050888 (PMC7278386; doi:10.3390/ani10050888)
Supplement: Supplementary file 1 [file animals-10-00888-s001.pdf]

Table S1 All exons sequences of porcine *RNF20* gene

Exon1:

AGGTTAGATCTCGGCCAAGGAAGGGACCAGACTGTGCGG**A(G)**CACCAGGAATCTCCACCTCCT
CCCGCTCCAGAGTCTCTCTCTGGGGCCTGC**C(A)**GTTGGCCTCGTCGGAGGCTGGGATCCTAGGCC
CCTCTCGAGTCGGATTGAGGCCCTTGTCCTGCCCCTCTCTCTCCATGCGAGGTCCCATCCAG

Exon2:

GAAAACAGCTATCATCTTGTGCCAAGATGTCAGGAATTGGAAATAAAAGGGCAGCTGGAGAGC
CTGGCACATCTGTGCCTCCGGAGAAGAAGACAGCTGTTGAAGATTCAGGGACTACAGTGGAAA
CAATTAAGCTCGGGGGTGTCTCTTCAACG

Exon3:

GAGGAGTTAGACATTCGAACACTGCAAACCAAAAATCGCAAACTGGCAGAAATGCTGGATCAA
CGGCAGGCCATTGAAGATGAACTCCGTGAGCACATTGAAAAACTGGAGCGAAGACAGGCCACC
GATGATGCCTCACTGTTGATTGTCAACCGGTATTGGAGTCAG

Exon4:

TTTGATGAGAATATCCGTATCATCCTGAAACGTTATGATCTGGAGCAAGGTTTGGGAGACCTACT
CACAGAAAGAAAAGCTCTTGTCGTGCCCGAGCCAGAACCAGACTCCGATAGCAATCAGGAACG
GAAGGATGAG

Exon5:

GGGAAGGGCAAGAGCCAGCTTTCTCTTTCCTTGCTACTTTGGCCAGCAGTTCCAGTGAAGAGAT
GGAGTCTCAGCTGCAGGAGCGTGTGGAATCCTCCCGCCGAGCTGTATCCCAGATTGTGACTGTCT
ATGATAAACTTCAGGAAAAAGTGGAGCTCTTATCACGGAAGCTCAACAGTGGAG

Exon6:

ATAATCTGATAGTGGAGGAAGCAGTACAAGAGCTGAATTCCTTCCTCGCACAAGAGAACATGAG
GCTTCAGGAATTGACAGACCTCCTTCAGGAGAAGCATCGCACCATGTCTCAGGAG

Exon7:

TTCTCCAAGTTGCAGAGTAAGGTGGAGACAGCGGAGTCAAGAGTGTCTGTCCTAGAGTCCATGA
TTGATGACCTGCAATGGGATATTGACAAAATTCGTAAGAGGGAACAGAGACTCAACCGACACTT
AGCAGAAGTCCTAGAGCGG

Exon8:

GTGAATTCGAAAGGTTATAAAGTATATGGTGCAGGCAGCAGTCTCTATGGTGGCACAATCACTAT
CAATGCCCGGAAG

Exon9:

TTTGAGGAGATGAATGCGGAGCTTGAGGAGAACAAAGAGTTGGCTCAGAACCGTCACTGTGAA
CTGGAGAAACTTCGGCAAGACTTTGAAGAAGTCACTGCACAAAATGAAAAGCTGAAG

Exon10:

GTGGAATTGCGAAGCGCAGTGGAGGAAGTGGTCAAGGAGACACCGGAATATCGCTGCATGCAA
TCACAGTTCTCTGTCCTATACAATGAGAGCCTGCAGTTGAAAGCACACTTGGATGAGGCTCGGA
CCCTGCTTCATGGCACCAGGGGGACCCACCAGCGCCAAGTTGAGCTCATTGAG

Exon11:

CGGGATGAGGTTAGTCTTCATAAGAAGCTGAGGACTGAAGTGATCCAGCTCGAAGATACACTGG
CCCAGGTCCGCAAGGAGTATGAAATGCTGAGGATAGAATTTGAGCAGACCCTTGCTGCCAATGA
ACAAGCAG

Exon12:

GCCCCATAAACCGGGAGATGCGTCACCTCATCAGTAGCCTCCAGAATCACAATCACCAGCTGAA
AGGGGAGGTTCTGAGGTATAAGCGGAAACTGAGAGAAGCCCAGTCTGACCTGAACAAG
Exon13:
ACTCGATTGCGCAGTGGCAGTGCCCTCTTGCAGTCTCAGTCCAGTACTGAGGACCCCAAAGAAG
AGCCTGCAGAGCTAAAACAAGATCCTGAGGACTTACCCGCCCAGTCCGCTACCTCGAAGACATC
TCAGGAGGAAGTCAATGAAATTAAGTCCAAAAGGGATGAGGAAGAGCGAGAACGAGAAAGGA
GGGAGAAAGAGAGAGAACGGAAAGAGAGCGGGAGAAGGAAAAGGAGAGAGAACGTGAGA
AGCAGAAACTAAAAGAGTCAGAAAAAGAAAGAGATTCTGCTAAGGATAAAGAGAAAGGGAA
ACATGATGATGGAAGGAAAAAGGAAGCAGAAATTATCAAACAATTGAAGATTGAACTCAA
Exon14:
GAAGGCACAGGAGAGCCAAAAGGAGATGAAACTATTGCTAGATATGTACCGCTCTGCCCCAAA
GGAACAGAGAGACAAAGTTCAGCTAATGGCAGCTGAGAAGAAGTCTAAGGCAGAG
Exon15:
TTGGAAGATTTGAGGCAAAGACTTAAGGACCTGGAGGATAAGGAGAAAAAAGAGAATAAGAA
AATGGCCGATGAGGATGCCTTGAGGAAGATCCGAGCAGTAGAGGAGCAGATAGAGTACCTGCA
GAAGAAGCTGGCCATGGCCAAGCAG
Exon16:
GAGGAAGAAGCTCTCCTCTCTGAGATGGATGTCACAGGCCAGGCCTTTGAAGACATGCAGGAG
CAGAATATCCGTTTGATGCAGCAGTTGAGGGAGAAAGATGATGCAAACTTCAAGCTCATGTCAG
AACGTATCAAATCCAATCAGATCCATAAACTGCTAAAAGAAGAGAAGGAAGAGCTGGCAGACC
AGGTTTTGACTCTCAAGACTCAG
Exon17:
GTTGATGCCCAGTTACAGGTGGTAAGAAAACTGGAAGAGAAGGAGCACTTGTTACAAAGCAAC
ATTGGCACAGGGGAGAAGGAGCTGGGTCTTAGGACCCAAGCCTTAGAGATGAATAAACGTAAG
Exon18:
GCAATGGAGGCAGCCCAGCTTGCAGATGACCTCAAAGCACAGTTGGAGTTGGCTCAGAAGAAG
CTACATGATTTTCAGGATGAGATTGTGGAAAACAGTGTCACCAAAGAAAAAGATATGTTCAATTT
CAAACGAGCCCAG
Exon19:
GAGGACATCTCTAGACTGCGAAGGAAGCTGGAGACCACAAAGAAGCCAGACAACGTACCCAA
ATGTGATGAGATTCTGATGGAGGAGATAAAGGATTACAAG
Exon20:
GCACGTCTGACCTGTCCCTGTTGCAACATGCGTAAAAAGGATGCTGTACTTACCAAGTGTTTTCA
TGTTTTCTGCTTTGAGTGTGTAAAGACCCGCTATGACACCCGCCAGCGCAAATGTCCCAAGTGCA
ATGCTGCTTTTGGTGCCAATGATTTCCATCGCATCTACATTGGTTGATCCAAGGCCAGAGAAGAC
GATGAGCTGGCTAGACAGGCACTTAGTCATTAACCACCAAACCTCTACCTCTTCTCTCCTTGACT
GTCACCTGCAAGGCAGTTTGTCTGTCGACTTCCTTTCCTTTGTAGACAGGTTGTCCTGTCTAATAG
CTAGATGACTTTGGGGGAAAGGACTGGTAATGCAAGTCTTGGGTTTTAGAATGAATTACAAACA
ACTTTTATTTGTCTCCTCTACCAGCTTTGTGTATTTCCTGCTTTTAGACTTTTTAGTATCTTCTTCAGG
CCCACTGTATAATCTTGGATTGTTCATTCCTCCTGAGGAAGTCAAATTGGTATTTGTGACACAGAG
AAAGGAACACATGGAGACACATGGCTACTTGTGGGTGATCTTTTAGAAATAAGCTGTCTACGAG
CATAAATAGTACTTGATTTGGGCAGTAAACTTTTTGTACACTTGGGTTATAAAATGGTATCATTGTG
CTGCTGTTTCCTGGATGGTTTGAAGCCTTAATGAAATTTATGTCCAGGATGATTCAGTCCATTTCTT
ATTGACTAGCTAAGTAATTTGTTAAGGTCAGCTGGCTTCCTTGTTATTTAAGTTTTAAATGTTCTGC

TCACTGATATCTTTAAATTTTGTGAGGCCAGAATAATTTCAAATACTATATCCCCTTTTATCTTATAA
TGAACTCACAATTGGAAAGGATGGCATGCAGGACTGAGATGGTAAAGAGATCTTCTGGGTGCTC
GGGGAACTTGTTCCAGGGAGGCCTTGGTGAGCTATGAATTACTCTCATCTGCCTGTCCACAGGGG
TTGTTGTGGCTCTTGTTTTCCAGAAGATTCTCTGTAATGTTTCTATTGGACTTTATACTCCACAAGC
TTCAATTAAAGCAGGATTCAGTT

Note: At the beginning and end of sequence, the grey shadow represented 5′-UTR and 3′-UTR, respectively. The initiation codon (ATG) and stop codon (TGA) were marked with yellow shadow in exon 2 and exon 20, respectively. The SNP1 (A-1027G) and SNP2 (C-975A) were shown in exon 1.
